# Collaborative Assessment and Management of Suicidality (CAMS) compared to treatment as usual (TAU) for suicidal patients: study protocol for a randomized controlled trial

**DOI:** 10.1186/s13063-016-1602-z

**Published:** 2016-10-03

**Authors:** Wenche Ryberg, Roar Fosse, Per Henrik Zahl, Inge Brorson, Paul Møller, Nils Inge Landrø, David Jobes

**Affiliations:** 1 Division of Mental Health and Addiction, Vestre Viken Hospital Trust, Lier, Norway; 2 Norwegian Institute of Public Health, Box 4404, Nydalen, N-0403 Oslo, Norway; 3 Blue Cross Norway, Storgata 38, N-0182 Oslo, Norway; 4 Department of Psychology, Clinical Neuroscience Research Group, University of Oslo, Oslo, Norway; 5 Department of Psychology, The Catholic University of America, Washington, DC USA

**Keywords:** Pragmatic randomized controlled trial, Suicidal patients, Treatment, Collaborative approach

## Abstract

**Background:**

Collaborative Assessment and Management of Suicidality (CAMS) is a therapeutic framework that appears promising to reduce suicidal ideation and suicidal cognition. CAMS has not previously been evaluated in a standard specialized mental health care setting for patients with suicidal problems in general. In this pragmatic randomized controlled trial (RCT) we will investigate if CAMS is more effective than treatment as usual (TAU) in reducing suicidal thoughts and behaviors. Effects will also be investigated on mental health and symptom relief in general and upon readmissions to inpatient units.

**Methods/design:**

The study is a multicenter, observer-blinded, superiority, two-armed RCT which will include patients from four clinical departments at Vestre Viken Hospital Trust, Norway. We aim to include 100 patients with moderate to strong suicidal problems, as defined by a score of 13 or more on Beck’s Scale for Suicide Ideation - Current. Patients are included regardless of diagnosis. Randomization will be performed using a stratified four-block procedure with treatment unit as the stratification variable. The duration of treatment will vary depending on patients’ needs and clinical assessments. Patients are interviewed by research staff at four checkpoints: baseline, 2 weeks, 6 months, and 12 months. Central outcome measures are the Beck Scale for Suicide Ideation - Current, Outcome Questionnaire – 45, and Suicide Attempt Self-Injury Count.

**Discussion:**

This pragmatic trial is effectuated within the Public Health Care System in Norway, where patients have multiple problems and diagnoses and therapists have a high work load. Results from this trial are highly generalizable to a typical everyday clinical setting, and one should expect similar results if CAMS is implemented in the future as a standard component in specialized mental health care systems.

**Trial registration:**

Open Science Framework: DOI 10.17605/OSF.IO/JHRM2. Registered 5 July 2015. ClinicalTrials.gov: NCT02685943. Registered on 8 February 2016.

## Background

By publishing its first World Suicide Report “Preventing suicide: a global imperative,” the World Health Organization (WHO) put suicide on the agenda worldwide [1]. The WHO’s goal is a 10 % reduction in the occurrence of suicide by 2020 through raising awareness, systematically mapping occurrences, and developing nationally tailored suicide prevention strategies within the general population and health services in particular. At a global level, approximately 800,000 people die by suicide each year, making suicide one of the leading causes of death worldwide. Suicide deaths, along with suicidal ideation and attempts, have a profound impact on society in the form of bereavement as well as on various public health issues and related economic factors.

In Norway, the suicide rate is 11.2/100,000; 500–600 lives are lost to suicide annually. In addition, attempted suicides are believed to be as many as 10 times higher or more than rates of completions. A national guideline for preventing suicide within specialized mental health services was published and gradually implemented since 2008 [2]. Despite the growing awareness both within public health and specialized fields such as mental health facilities, the rates of suicide remain relatively stable dating back to 1996 [3].

Mental illness is a known risk factor for suicide; affective disorders, psychosis, substance abuse, and personality disorders are commonplace among suicide completers. It is estimated that as many as 45–90 % of those who die by suicide suffer from mental illness [4, 5]. Suicide-related presenting problems are involved in 50–70 % of all admissions to specialized mental health care services in Norway [6, 7]. Concomitantly, people who have received inpatient treatment in the mental health care services are especially vulnerable. The first weeks following discharge are notably associated with increased suicidal risk [8]. Accurate risk assessment and effective treatments for at-risk patients are imperative.

As people suffering from mental illnesses seem to be disproportionally overrepresented in the suicidal fatality statistics, it is essential to develop effective treatment models. However, remarkably little research exists on which treatments are the most effective, and evidence-based treatment models for suicidal patients are rarely disseminated in clinical practice [9]. Most suicidal patients within mental health care are treated with therapies that are not yet empirically validated for effectively treating suicidal risk [10]. There are a few exceptions, however. For example, dialectical behavior therapy (DBT) has been effective for suicidal patients with emotionally unstable (borderline) personality disorder [11]. Likewise, cognitive therapy for suicide prevention (CT-SP) has received support as an effective treatment in preventing suicide attempts for adults who recently attempted suicide [12, 13]. For more information, see a recent review on effective psychological treatments for suicidal risk by Jobes and colleagues [14].

For suicide-related problems, a small set of novel approaches have been developed. Among these promising approaches is the “Collaborative Assessment and Management of Suicidality” (CAMS) for assessing and treating suicidal patients using a problem-focused suicide-specific approach [15]. CAMS is a therapeutic framework that emphasizes collaborative assessments of a patient’s suicidal state and a problem-focused treatment that centers on patient-defined suicidal “drivers” (those problems that make suicide compelling to the patient). To date, CAMS is supported by a range of clinical treatment studies including seven correlational trials and two randomized controlled trials (see Andreasson et al. [16]; Jobes [17]; Comtois et al. [18]). As such, CAMS may have the potential to address a common critique raised against clinical suicide prevention efforts both in Norway and internationally: there has been a disproportionate focus on mechanical risk assessments at the expense of developing and conducting effective suicide-specific treatments [19, 20]. The current trial is one initiative that may contribute to the further development within the field of clinical suicidal prevention by comparing CAMS to “treatment as sual” (TAU) within a randomized controlled trial for suicidal patients in Vestre Viken Hospital Trust.

### The CAMS approach

CAMS is a psychotherapeutic framework developed by David Jobes [15]. As a semi-structured therapeutic framework, CAMS is guided by both a philosophy and specific therapeutic strategies that amplify active collaboration between the patient and the therapist, while assessing and treating the subjective underlying factors or drivers of suicidality [21]. Moreover, CAMS is designed to create a strong clinical alliance increasing the patient’s motivation to want to live.

Within the CAMS approach to suicidal risk, there is the central use of a multipurpose assessment, treatment planning, tracking, and outcome tool called the Suicide Status Form (SSF). In brief terms, the SSF uses both quantitative and qualitative assessment ratings of five central suicide markers previously described by Shneidman, Beck, and Baumeister: psychological pain, stress, agitation, hopelessness, and self-hate. In addition to rating these constructions on a 1–5 rating scale, the patient is encouraged to describe the qualitative aspects of each entity in such a way that the problem areas that contribute to suicidality are clarified. Further on in the initial CAMS assessment, the patient identifies reasons for living and reasons for dying, as well as his/her wishes to live and wishes to die. The SSF has various formats: for the first session, all interim sessions, and the outcome/disposition session. In every CAMS session, however, the dyad begins with assessment of the key markers and ends with a side-by-side seating to review and update both the stabilization plan and the driver-oriented treatment plan. As a part of the CAMS approach and structure, the patient and therapist collaboratively develop a suicide-specific treatment plan that continues to identify, target, and treat the drivers of the patient’s suicidality. CAMS is described as “non-denominational,” which means the framework can be used across disciplines and can be effectively used transtheoretically as long as the provider follows the philosophy and clinical structure indicated by the SSF format. CAMS clinicians are empathetic of suicidal states and pursue suicide-specific treatment in a supportive, collaborative problem-solving, non-judgmental manner.

There is fairly robust and replicated evidence that CAMS is superior to other approaches for suicidal ideation and suicidal cognition [17, 22]. The overall CAMS approach has received preliminary support in seven non-randomized outcome studies. These include various correlational studies that support the usefulness of the approach in both outpatient and inpatient settings [23–25]. To date, well-powered randomized controlled trials are limited, but one small RCT conducted by Comtois and colleagues showed CAMS to be superior to TAU in terms of reduced suicidal ideation and symptom severity and increased hope, retention, and superior patient satisfaction [18]. A new Danish superiority RCT compared treatment conditions for borderline personality disorder with self-harm and suicidal behavior [26]. In this somewhat underpowered RCT, the investigators found no significant difference between DBT and CAMS for self-harm and suicide attempts at 28 weeks following treatment, which was surprising, given the robust evidence of the effectiveness of DBT for self-harm and suicide attempt behaviors [16].

#### Aims and research questions

Our aim is to investigate whether CAMS represents improved services to patients with suicidal ideation and elevated suicidal risk in comparison to existing practices. The primary objective is to investigate whether treatment for suicidal patients by using CAMS results in a clinically and statistically significant reduction in suicidal thoughts and behaviors as compared to treatment as usual (TAU). Secondary aims are to investigate effects of CAMS and TAU upon mental health in general, symptom relief, and readmissions. Furthermore, we will test the influence of various moderating variables upon outcome, including qualities of the working alliance, the patients’ initial level of self-efficacy, and substance and alcohol abuse.

## Methods/design

In a pragmatic RCT, CAMS will be compared with TAU in the department of mental health and addiction, Vestre Viken Hospital Trust, Norway. Vestre Viken Hospital Trust consists of several formerly independent hospitals and institutions, covering 26 municipalities with a population base of 470,000 people. The trial will be conducted at four different outpatient and inpatient treatment units situated in four different geographical areas. At Baerum District Psychiatric Center one general inpatient facility and one acute inpatient facility participate in the trial. One acute in-ward facility at a psychiatric hospital in Asker (Blakstad Hospital) also attends the trial. Furthermore, in Drammen District Psychiatric Center one acute ambulatory outpatient unit and one team within the general outpatient clinic participate. Lastly, at Kongsberg District Psychiatric Center two units are involved: one acute inpatient ambulatory team and one team from the general outpatient clinic. Together, the participating inpatient units admitted approximately 850 patients for hospitalization in 2015. The outpatient clinics saw approximately 2000 patients for treatment. Information is not available regarding the proportion of those treatment courses that were associated with suicidality. However, as reported by Ruud in a representative study, up to 70 % of those seeking acute psychiatric health care in Norway suffer from some degree of suicidal-related problems (thoughts, plans, actions) [7]. Similar figures were reported by Mellesdal and colleagues [6].

### Eligibility criteria

Suicidality can be viewed as a transdiagnostic issue that often increases with symptom severity. Hence, broad inclusion criteria are meaningful in our clinical setting.

#### Inclusion criteria

- The inclusion criteria are as follows:
- Newly referred adults above 18 years of age
- Symptom severity and complexity that require health care and treatment within specialized care in Norway
- Ongoing moderate to severe suicidal thoughts, plans, or actions within the past 2 weeks, qualifying to a score of 13 or more on the Beck Scale for Suicide Ideation - Current (BSSI-C)
- Ability to provide informed consent

#### Exclusion criteria

The exclusion criteria are as follows:

- Age less than 18 years
- Symptom severity and complexity beneath the threshold that elicits rights to necessary health care within specialized care in Norway
- Known or previously diagnosed intellectual disability or dementia
- Known or previously diagnosed developmental disorder such as autism or Asperger’s syndrome
- Ongoing psychosis in terms of active hallucinations and delusions which hamper the ability to provide informed consent
- Poor Norwegian acquisition which requires a professional translator during the research interviews or the therapeutic process
- Previous exposure to CAMS treatment components

The main principle that we follow is that project participants may be included if the symptoms are above threshold as measured by the BSSI-C. The role of informed consent for participation is detailed in the section “Ethics approval and consent to participate.”

The electronic patient journal contains information regarding previous diagnosis and is accessible to the research team and aids the inclusion/exclusion process. If research assistants or clinicians in companionship suspect that an eligible participant has a developmental disorder or intellectual disability, the participant is not recruited into the project for further screening but follows usual treatment procedures in the clinic. If such information or diagnosis is made post hoc after inclusion, the participant will be excluded from further participation. Whether Norwegian language acquisition level is sufficient for inclusion was pragmatically decided to be a clinical judgment, whereby participants with need of a translator during assessment or therapy were excluded. If a patient presents with active psychotic symptoms in terms of hallucinations or delusions, she/he is not invited to participate. If such functioning is identified during the screening process (e.g., by MINI), the patient will be excluded from the trial and will continue with ordinary treatment in the clinic.

Finally, we exclude patients with previous exposure to CAMS treatment components, most notably patients who previously have been administered the SSF. As the entire hospital shares the same electronic patient journal system, we are able to check this before recruiting patients. Use of antidepressants or other kinds of medications is permitted; however, we will register and map their occurrence.

### Power calculation

In calculating power, we use one-sided confidence intervals because previous studies have indicated CAMS to be superior to TAU [18, 23–25]. We use the following assumptions: (1) the main outcome variable BSSI-C score after treatment — which is scored on a 0–38 point scale — has a standard deviation of 7 points [27]; (2) there are equal sample sizes in the experimental and control groups; (3) a four-point difference in BSSI-C scores at follow-up will represent a meaningful clinical difference between CAMS and TAU; and (4) the alpha level is 0.05. Based on these assumptions, 82 % power will be achieved by including 80 participants in the trial, who will be randomized to CAMS or TAU. Assuming a 20 % drop-out rate, our desired sample size is 100 participants at inclusion.

### Procedures

#### Recruitment

Recruitment is a collaborative process between the research assistants and general clinicians. Clinicians and leaders in the clinics are informed about the project and encouraged to inform and invite eligible patients to participate. Furthermore, four research assistants monitor the patient flow by attending meetings where the general patient flow is handled and processed: morning meetings where newly admitted patients are discussed, intake meetings where referrals for treatment are processed, and other fora where patient referrals are discussed. When potential participants are identified by clinicians or research assistants, the clinician who conducts the first assessment with the patient informs the patient about the project and invites him or her to participate if suicidality is part of the current problem complex. A short invitation letter is compiled for this use. If the patient agrees, he or she will meet with a research assistant to receive detailed information about the project. If the clinician finds that the patient no longer struggles with suicidal-related problems, e.g., the referral letter was misleading, the patient is not invited to participate. Furthermore, if the clinician in the first assessment finds that the patient’s language acquisition requires the use of a translator, she/he will not invite the patient to participate. A close collaboration between the clinic and the research team is essential.

Interested patients will meet a project assistant who provides thorough and written information about the aim of the study, the study design, and procedures. Ethics, data collection and storage of information, confidentiality, and the possibility to withdraw from the project at any time are also covered in these meetings. All screening interviews with research assistants will be conducted at the clinical unit already familiar to the patient. If an informed consent is offered by the patient, the research assistant follows up with the inclusion screening interview BSSI-C. Eligible participants who are actively suicidal as indicated by a positive score on item SS104 or SS105 and who score 13 or more are accepted for inclusion. At this point the patient is informed whether he or she may be included, and the randomization procedure is performed. Following this, the remaining baseline measures are acquired (see Table 1). Those with scores below the BSSI threshold are excluded from the trial but will receive usual care within the clinic.

**Table 1:** Measurements at follow-up

Measures	T1 baseline	T2: 3 weeks	T3: 6 months	T4: 12 months	Outcome (1–10 years)
Primary outcome measure					
Beck Scale for Suicide Ideation - Current (BSSI-C)	x		x	x	
Secondary outcome measures					
Suicide Attempt Self-Injury Count (SASIC)	x		x	x	
Outcome Questionnaire-45 (OQ-45)	x		x	x	
Norwegian Patient Registry data (specified below)					x
Potential moderators and descriptive measures					
MINI		x			
Structured Clinical Interview for ICD-10		x			
Treatment history interview - short form	x		x	x	
Generalized Self-Efficacy Scale (GSE)	x		x	x	
Working Alliance Inventory - Short Revised (WAI-SR Client)		x			
Alcohol/Drug Use Disorders Identification Tests (AUDIT and DUDIT)	x				

#### Randomizing and treatment allocation

**Fig. 1:**
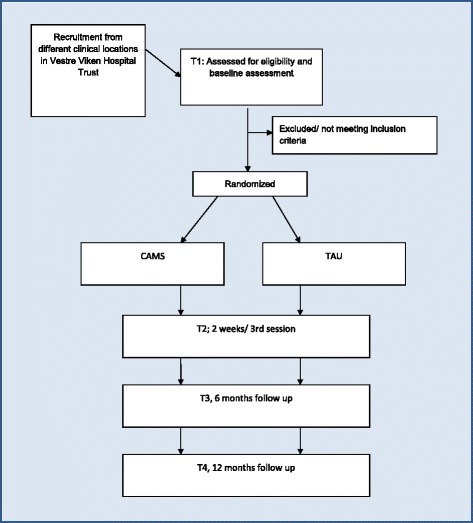
Flowchart and timetable

Allocation to treatment group will be performed using a stratified four-block randomization procedure with treatment unit as the stratification variable. The randomization will be concealed to the clinicians recruiting patients to the study and is carried out by an independent statistician at the Norwegian Institute of Public Health. After screening and randomizing, the study participants will be followed for one year and meet with a trained research assistant at four checkpoints: T1, T2, T3, T4 (see Fig. 1).

#### Assessments and measurements

The test battery will be administered by four research assistants. Completion of the baseline and the first follow-up interview (T1, T2) will take approximately 50–70 minutes each. Follow-up screenings after 6 and 12 months each will take approximately 25–45 minutes. We will perform a screener crossover at T3 and T4 so that the research assistant who screens for follow-up is blinded for treatment condition. Treatment condition is not concealed or blinded for the patient or the clinician treating the patient.

The research assistants consist of experienced psychiatric research nurses and psychiatric research health workers and the first author (WR). All have been formally trained for conducting the diagnostic interviews, and one research nurse has previous experience with attending and screening in another research project. The diagnostic interview is conducted in the follow-up interview at T2, after inclusion. After conducting the clinical diagnostic interview, the research assistant discusses and clinically validates the diagnostic conclusion with the first author or with the experienced research nurse. Beyond this, a more formal reliability check on diagnosis will not be carried out. Data will be stored in paper form until all follow-up interviews are completed and then entered into digital form for statistical analysis.

#### Measures

We have kept the set of research measures at a minimal level, without losing meaningfulness and thoroughness. All included measures are widely used within the psychiatric clinical and research field, showing acceptable validity and reliability and good overall psychometric properties (see Table 1 for an overview of measures).

#### Primary outcome

Beck’s *Scale for Suicide Ideation* - *Current (BSSI-C)* is an interview scale with 19 items that measures a patient’s suicidal ideation at its worst point during the past 2 weeks [27]. Items are scored on a Likert scale ranging from 0 to 2, where high scores indicate severe suicidal ideation. This scale has been found to be a valid and reliable measure of suicidal ideation for use with psychiatric patients, including test-retest reliability and internal consistency scores above .90 [27]. BSSI-C will be the primary inclusion measure (see above) and will be administered at baseline (T1) and at 6 and 12 months (T3, T4).

#### Secondary outcomes

The * Suicide Attempt Self-Injury Count (SASIC)* is a brief interview covering past self-inflicted injuries, categorizing them into suicide attempts and non-suicidal acts [28]. The tool also creates counts of self-inflicted injuries by method, medical risk severity, and lethality. It has a Lifetime form and a Recent form which covers the last 6 months. The Lifetime version will be provided at baseline and the Recent form will be administered at T3 and T4.

The *Outcome Questionnaire - 45 (OQ-45)* is a 45-item questionnaire designed to measure key areas of mental health functioning (symptoms, interpersonal problems, and social role functioning). It is a widely accepted tool for identifying, tracking, and measuring behavioral health treatment outcomes and will be the measure of symptom distress in this study. OQ-45 possesses good psychometric properties when used with adult psychiatric patients, and it was recently shown to have high reliability and concurrent validity in a Norwegian sample [29]. OQ-45 will be administered at baseline and after 6 and 12 months.

*Norwegian Patient Registry (NPR)* measures are taken from administrative data on patients*.* All patients admitted to a hospital in Norway are registered in NPR. Primary NPR measures will be suicide attempts, new hospital admissions to any health treatment, and discharge diagnoses entered for the readmissions. Secondary measures will be intoxication and death by any cause.

#### Potential moderators and descriptive measures

The *Mini International Neuropsychiatric Interview (MINI)* will be used at baseline to identify DSM-VI and ICD-10 disorders associated with mood and psychosis along with other disorders. Studies have shown a good reliability and validity compared with the CIDI and the SCID-I interview [30]. In addition, the Structured Clinical Interview for DSM-IV Axis II Borderline Personality Disorder (SCID-II) will be administered to identify patients with borderline personality disorder given the importance of this group in the suicidality literature [31].

The *Generalized Self-Efficacy Scale (GSE)* is a reliable and valid measure comprising 10 items assessing the strength of an individual’s belief in the ability to respond to novel or difficult situations and to deal with a large variety of stressors [32, 33]. The GSE will be administered at baseline and after 6 and 12 months.

The *Working Alliance Inventory - ShortRevised (WAI-SR)* is a 12-item measure that will be used to assess the patient’s and therapist’s alliance after their third session together [34]. We will use the patient version of the instrument, which has high internal consistency and correlates well with other alliance measures.

Substance abuse is assessed with the *Alcohol Use Disorders Identification Test (AUDIT)* [35] and the *Drug Use Disorders Identification Test (DUDIT)* [36]. The AUDIT includes 10 questions about patterns of alcohol use and dependence. The DUDIT includes 11 questions about use or abuse of a list of drugs. Both instruments have satisfactory psychometric properties in clinical and non-clinical samples, with overall reliability above .80 and convergent validity, sensitivity, and specificity above 85 % [37, 38]. AUDIT and DUDIT will be scored at baseline.

The *Suicide Status Forms (SSFs)* are administered (initial, tracking, and outcome versions) as part of the course of care in CAMS (used for CAMS condition only). The SSF has six core rating assessments derived from theoretical approaches of Shneidman, Beck, and Baumeister and has well-established reliability and validity [39, 40].

As two of the measures (SSI-C and SASIC) did not have a Norwegian version, they were translated according to conventional procedures.

### Interventions

#### CAMS intervention

All patients randomized to the CAMS arm of the trial meet an adherence approved CAMS therapist. In brief terms, CAMS is a supportive psychotherapeutic approach that targets suicidality as the primary focus of assessment and treatment. CAMS treatment follows a predefined structure, where the SSF multipurpose assessment tool is routinely employed. A typical treatment course is characterized by employing weekly sessions of 50–60 minutes duration. Every session is initiated by filling out the SSF in a side-by-side manner. The SSF assesses and tracks the treatment process and highlights central suicide markers: psychological pain, stress, agitation, hopelessness, and self-hate (see the subsection “The CAMS approach”). In the first session, approximately 20 minutes are used to fill out the first part of the SSF as part of a “core assessment.” During the interim sessions, approximately 5 minutes are used to fill out this part of the SSF. A problem-specific treatment plan addressing the patient’s suicidal drives is jointly developed during the first session and routinely evaluated in the last part of each CAMS session. Furthermore, as part of CAMS care, a stabilization plan is developed during the first CAMS session and evaluated and improved during every consecutive session in order to increase the patient’s coping skills. Following the first assessment, various suicidal “drivers” are identified and treated during the CAMS course. Ongoing CAMS care consists of developing adequate coping skills (through the stabilization plan) and helping the patient identify and cope with their drivers of suicidality in a problem-oriented way. In CAMS, suicidal drivers are divided into two categories: direct and indirect drivers. Direct drivers are thoughts, feelings, or behaviors that increase specific suicidal thoughts or feelings. Indirect drivers are factors that themselves do not produce suicidal states but instead increase the vulnerability to such states (e.g., unemployment, economic difficulties, trauma from the past).

There are no mandatory homework assignments during a CAMS course. The duration of CAMS care is dependent on the treatment progress and varies between patients. Treatment is concluded when adaptive coping skills are developed while the patient scores him/herself below 3 on subjective suicidal risk on the SSF during three consecutive CAMS sessions. Clinicians in the inpatient acute ward have reported that this criterion implies extended inpatient stays in the clinic, which is deemed unacceptable. Therefore, a deviation from the manual is necessary. However, when recruiting from the inpatient acute wards, we will aim to include those who are believed to stay for longer than only a couple of days and only those who are believed to be able to have at least three sessions with their allocated clinician after randomization. We will analyze all patients who have received three or more CAMS sessions.

#### Implementing CAMS and adherence training

Recruitment of project therapists began in the spring of 2014; the project is continuously including and training new therapists when needed. We have trained CAMS therapists by applying two different models. Initially, we offered a traditional workshop training program, but later experienced the need for extended training possibilities. Flexibility is important, and we continuously need to be able to offer training due to natural therapist turnover in staff (see Table 2 for a summarization of learning steps in the two models).

**Table 2:** CAMS training

	Teaching philosophy and strategies	Skills training, role play	Shared literature	3 videotaped supervised therapies	Acceptable scores on the CAMS Rating Scale
2 days traditional workshop	✓	✓	✓	✓	✓
1 day e-learning and 1 day workshop	✓	✓	✓	✓	✓

The two first groups were trained at a 2-day introductory workshop in the CAMS theory, philosophy, and strategies by a Danish clinical psychologist and approved CAMS trainer. The workshop emphasized and facilitated in vivo training of the CAMS strategies through group work where the participants joined up in pairs and exercised new skills in role play. Other therapists who joined the project later have instead followed the online training program in CAMS with American experts. First, these therapists completed the e-learning course (which requires approximately 1 work day) in a self-paced manner and then met in a 1-day workshop with other e-learners and a CAMS supervisor. During the workshop, the CAMS philosophy and strategies were reviewed, and the group practiced CAMS strategies using role playing and interview techniques. In addition to a selection of CAMS-related teaching material, all trainees have received a copy of the CAMS treatment manual as well as Jobes’ book *Managing Suicidal Risk: A Collaborative Approach* [15]*.*

To be approved as a project therapist in the CAMS group, the therapists must conduct three videotaped training therapy sessions following the introductory training. All training therapies were rated by project staff according to the CAMS Rating Scale (CRS), which contains 14 statements covering three main areas of competency: “therapeutic philosophy,” “session framework,” and “overall rating.” Each statement is scored on a 7-point Likert scale. In order to qualify, a mean score of 3 or “satisfactory” (3 or more) was required on the CRS. Of 30 potentially eligible therapists who completed CAMS training, 14 therapists completed the adherence training. Those who completed adherence training met the requirements on the CRS to be approved as a CAMS therapist. Nonetheless, currently only 9 are actively involved and treat recruited patients in the study. After adherence approval, the CAMS therapists are offered supervision from the first author (WR) upon request.

#### TAU intervention

All patients included in the trial receive treatment within specialized care in our clinic. The TAU intervention consists of psychotherapy from diverse theoretical orientations such as psychodynamic, cognitive, or eclectic orientations, combined with psychopharmacological treatment as needed; these treatments are not manualized. A typical outpatient practice is weekly sessions with a duration of 45 minutes. In times of crises, increased follow-up frequency is usual to prevent an unnecessary hospitalization.

To enhance external validity in the study, therapists in the TAU group receive no training or supervision from the project, as they are expected to continue their clinical practice as usual. However, TAU is not “uncontrolled,” and both treatment interventions must adhere to central national guidelines. Health care within specialized treatment centers in Norway is governed by several central legislations, including The Health Personnel Act, the Patients’ Rights Act, and the Specialized Health Services Act. Furthermore, national treatment guidelines are developed regarding specific diagnosis such as attention deficit disorder, substance abuse and addiction, bipolar disorder, psychotic disorders, and depression. A central guideline that is especially relevant in this context is the National Guideline for the Prevention of Suicide in Mental Health Care in Norway [2]. This guideline is implemented in most inpatient and outpatient clinics and ensures a safety focus. According to the guideline, all patients in psychiatric care are to be asked whether they have suicidal thoughts. A formal mapping and assessment of suicidal risk by competent health workers is recommended when the patient is at risk or when he or she confirms suicidal-related problems. Generally, these guidelines describe, summarize, and recommend evidence-based treatment interventions. However, there are no fixed predefined or obligatory theoretical orientations or treatment programs demanded or expected locally or nationally.

TAU therapists in our study have diverse theoretical preferences, as the local units are not devoted to any specific orientation. Since the various theoretical orientations and supervision in TAU are unknown to the project, we will conduct a survey where this is probed. Lastly, a new initiative — a 5-year program termed “The Norwegian Patient Safety Programme: In Safe Hands” — aims to reduce patient harm and improve patient safety in Norway [41]. Suicide prevention during acute inpatient admittances is a prioritized area, and a range of measures are under continuous national implementation: The patient is to meet a specialist for assessment within 24 hours after hospitalization, rooms and surroundings at the wards are secured, and standardizing of assessment of suicidal risk at transition situations is described. Furthermore, the safety program recommends actions at the point of discharge to prevent patient harm such as developing a crisis plan, the involvement of relatives/next of kin, and the scheduling of an appointment within a week after discharge with a health professional when needed.

### Therapists’ backgrounds: CAMS and TAU

All therapists in CAMS and TAU are skilled psychologists or psychiatrists; some are specialists in their field. Thirty therapists (CAMS and TAU) are currently actively participating in the study (see Table 3). The CAMS therapist group now consists of nine adherence approved participants, where eight are psychologists and one is a psychiatrist. Four are specialists in their field of expertise and have more than 10 years of clinical experience. Among the 21 therapists in the TAU group, 6 are psychologists and 5 are psychiatrists. Further, 3 additional residential physicians participate in the TAU group. In addition, within the TAU group, an interdisciplinary team with 7 experienced nurses and social workers is part of the treatment team at sites that have acute ambulatory services. Together with a responsible psychologist or psychiatrist, this group functions as the patient’s therapist within the frame of an acute ambulatory practice. All therapists were self-selected.

**Table 3:** Therapists’ backgrounds

	Psychologists	Doctors/residents in psychiatry	Ambulatory team- psychiatric nurse	10 years of experience or more	Specialists in clinical adult psychology	Psychiatrists/chief attending physicians
CAMS: 9	8			4	3	1
TAU: 21	6	3	7	11	3	5
SUM: 30	14	3	7	(15)	(6)	6

#### Removal from the trial, safety monitoring, and acceptability

All participating therapists and research assistants are health care professionals with considerable experience in the treatment and care of psychiatric patients with suicidality. With the consent of the patient, a brief report about symptom severity and diagnostic considerations obtained from the research interviews at T1 and T2 will be documented in the hospital electronic patient journal. To avoid confusion of roles and responsibility, all clinicians are reminded about their obligation to independently assess suicide risk and diagnostic assessments.

Non-responders or patients with high symptom severity (CAMS and TAU) who have been discharged from treatment at follow-up will be encouraged to contact their general practitioner for assessment and possible re-referral to treatment. Furthermore, patients with active suicidal ideation will be cared for by hospital staff or transported to an emergency room if necessary. The CAMS (or TAU) therapist is independently responsible for safety issues and will terminate CAMS (or ongoing TAU) treatment if he or she assesses the process to be insufficient or harmful. In such cases the therapist reports this information back to the research assistants. Non-responders in the TAU group are not offered subsequent CAMS treatment.

CAMS previously has been shown to be an effective treatment model [16, 17, 22]. In a pilot study from 2012, Ellis and colleagues found that CAMS was successfully implemented and accepted by both patients and clinicians within the frameworks of inpatient settings [24]. Also, in a Danish outpatient naturalistic study, CAMS was found to be feasible to implement as well as effective for reducing suicidal ideation in ordinary clinical care [25]. Patients reported that the therapeutic CAMS sessions were the essential factor for their improvement. In an online survey where adherence to CAMS treatment components was evaluated, Crowley and colleagues reported that CAMS treatment philosophy and strategies were successfully adopted, considered comfortable, and used with confidence by a large majority of 120 clinicians [42]. On this basis we expect that CAMS will probably be acceptable to the patients and clinicians within our clinic.

### Procedures to prevent treatment leakage or contamination

The project collaborates closely with the clinical units and teams participating in the trial. Leaders and clinicians are informed about the study design, purpose, and procedures as well as the importance of not contaminating the study by offering TAU participants CAMS treatment. CAMS training and supervision is offered to eligible therapists for the project only. Those who did participate at the training courses are instructed not to deliver CAMS treatment components to any patient who might be a potential participant to the trial in the future. Clinicians who have attended CAMS training and who for some reason are not participating in the project as CAMS therapists are not accepted in the TAU arm.

Furthermore, we ensure that all clinicians are informed about the trial through a system for “critical information” in the electronic patient journal system that is common to all clinical units. Each time a project participant’s journal is opened, a dialogue box appears and informs about his/her attendance in the trial and that CAMS treatment components are not to be added — especially not the SSF. Of note, three inpatient units have used the SSF as a screening tool for assessing suicidality. Two of these units are not attending the project, and the third unit has discontinued this practice before being accepted as a participating unit in the trial. Consequently, some former patients who seek treatment after a relapse may previously have been screened with the SSF. These patients are excluded and not accepted as trial participants. This is routinely controlled by the first author and one of the research assistants. Lastly, CAMS therapists may not consult patients in the TAU condition.

### Statistical analysis

We will use generalized linear regression to examine the effects of treatment condition (CAMS versus TAU) upon suicidal thoughts (SSI-C scores) and mental health functioning (OQ-45 scores). Possible moderator effects will be estimated by using an array of covariates, including baseline measures (age, gender, ethnicity, diagnoses, and psychological measures), working alliance, medication during the treatment period, varying treatment length, new treatments delivered during follow-up, and an eventual leakage of CAMS elements into TAU.

Statistical analysis will be by intention to treat. To manage and replace missing data, we will create several sets of plausible imputed data, based on the Bayesian approach, by using multiple imputation procedures for the variables of interest, following the guidelines of Sterne and colleagues [43]. In a further sensitivity analysis we will perform the analysis using the per protocol method, excluding patients who did not complete treatment or missed measurements.

### Ethics

The trial has been approved by the ethical committee of medical research, health region South East Norway (reference number 2014/465). Several ethical considerations must be discussed and handled when conducting research on a vulnerable patient group. First, there is no reason to believe that CAMS has a poorer effect than TAU, as evidence until now suggests otherwise. Moreover, we have no indication that some patient groups respond better or worse than others. It follows that the CAMS arm of the trial will offer equally effective or better treatment as compared to TAU. The main intention of the study is to evaluate whether CAMS is more effective than TAU. Indeed, the major problem in this field is that the treatments that are administered lack controlled research support. If our hypothesis is supported, our goal is to contribute to the use of CAMS for future patients in our own and other clinics. It is important to stress that all suicidal patients will receive treatment during the study, whether CAMS or TAU. We will not offer a control group of patients who receive no treatment or postponed treatment. Participation is voluntary, and the patients are informed of their ability to withdraw from the study at any time.

The patients attending the project are not offered any remuneration, such as payment or gifts, for their participation in the trial. Therefore, we expect little to no vicarious motives for attendance other than general openness, agreeability, and interest. However, as we experienced recruitment difficulties, we decided to offer a small token of appreciation (a bag of chocolates) worth 6 US dollars to the clinical units for each patient they recruited to the trial.

The trial has been preregistered in the Open Science Network, registration code DOI 10.17605/OSF.IO/JHRM2 and in ClinicalTrials.gov with the ID NCT02685943. We have committed to publishing any results from the study and consider any findings important information in our work towards improving health services for patients who are suicidal.

## Discussion

Randomized controlled trials are generally considered to be scientifically superior for evaluating treatment effects. The unique random allocation to treatment conditions controls for potential confounding variables, so that it is plausible to infer that intergroup variability in outcomes is caused by treatment conditions [44]. However, as noted by Cochrane some 40 years ago, “between measurements based on RCTs and benefit … in the community there is a gulf which has been much under-estimated” [45]. That is, a main criticism of the RCT is not the procedure in itself but rather the problem of generalizability and external validity [46, 47]. The important issue is that knowledge from RCTs should be applicable outside idealized research settings, so that it can be used to guide decision making in real-life clinical settings [48].

A distinction between efficacy and effectiveness trials, or explanatory and pragmatic trials, has been suggested. Some recommend that researchers more often conduct effectiveness trials [48, 49]. For our study, we decided that a pragmatic trial with focus on effectiveness would be preferable, as it maximizes clinical relevance. During the process of designing and conducting the trial, pragmatic values have guided our general decision making. An effectiveness trial aims to unfold whether an intervention could work within normal practice. In comparison, an efficacy trial operates within favorable and ideal research settings where participants are highly selected and adherence to treatment conditions is closely monitored and controlled. Our pragmatic trial is all about our patients, our practice, our culture, and our clinical reality. It is these topics about which we wish to generate more knowledge. The choice of applying broad inclusion criteria is one demonstration of pragmatism. This mirrors the usual clinical reality: patients seeking help in the mental health system often have multiple problems. We expand the general applicability of the results from the trial by making ongoing suicidality the main inclusion criterion independent of the patient’s clinical diagnosis. Further, by using a conservative and limited test battery, we enhance general feasibility in the trial process. However, as repeated symptom measures are not routine practice within our services today, an independent effect may be expected that may limit generalizability. However, this factor should not cause systematical differences between the outcome for TAU and CAMS.

All in all, this trial demands little to no structural changes within the organization. We therefore argue that if the results are positive, implementation of the CAMS model is highly realistic, and one should be able to expect similar effects in a standard clinical setting independent of any concomitant research project. However, we do experience a range of challenges. The main concerns are associated with the setting, recruitment, and possible selection bias, as discussed below.

### Setting

The unique setting of the pragmatic trial generally offers important challenges that may affect motivation of attendance for the involved therapists and also cause recruitment difficulties. For the involved therapists, participation in the study will usually represent an assignment “on top” of daily demands and expectations. Furthermore, as the project is spread out across several units within the clinic, with low representation at each site, the project therapists may experience little co-ownership and companionship in the project as a whole. There are few meeting points with other participants and no possibilities for reducing other work assignments. In this, the study mimics the often lonely and independent reality of a therapist within the mental health services. Low representation of CAMS therapists at each site combined with the need to perform the somewhat unpredictable randomization procedure complicates the inclusion process.

These factors as a whole may hamper clinicians’ motivation and ability to participate actively in the range that we expect, as we rely on each therapist’s inner motivation to take part in the project. In order to meet these challenges, we find it important to continuously motivate and support the participating therapists. Further, continuous communication and anchoring of the project at all levels of leadership as well as persistent offering of information about the project and its possibilities are important. On the other hand, as there are no strong secondary gains to achieve by participating in the study, we expect the research data and results to have high ecological validity.

### Recruitment

Recruitment difficulties are a substantial problem encountered in many trials. Our experience so far is that patient motivation for attendance is only a small hindrance and seems manageable. Instead, the recruitment challenge seems to be more pertinent for the therapists, regarding their motivation and ability to perform the randomization procedure. Such a reluctance to commit to a research project is not novel or exceptional and has previously been described for other trials. Typical therapist challenges that have been reported include time constraints, lack of training, concerns about the therapeutic alliance/relationship, confusion about inclusion criteria, concerns regarding loss of clinical autonomy, lack of rewards [50], misconceptions about the trial, and paternalism [51].

Our recruitment difficulties are multifaceted. Strategic and historical factors in the developmental stage explain part of the picture. At the first stage of the project, we planned to conduct the trial at outpatient facilities only. We planned this, first, because this is where the majority of patient treatment is conducted, but also because hospital units systematically are reorganizing services in such a way that inpatient clinics are scarce, with only short and abrupt patient stays. However, after training the first CAMS therapist group from mainly outpatient facilities, we experienced difficulties concerning adherence training. Therapists reported that eligible patients were *simply not there* but received treatment at other sections (inpatient facilities and newly opened acute out-ward facilities) within our services. Further, clinicians argued that when the patients were discharged from the acute in-ward facilities, suicidality issues seemed no longer to be clinically relevant or meaningful to focus on in therapy. These concerns demanded a reorientation. During the fall of 2014 and winter of 2015, the second and third CAMS courses were completed, this time with therapists working mainly in acute and inpatient clinics. Obviously, the trial depends on being located in parts of the system where suicidal patients receive treatment.

Recruitment difficulties (and solutions) cannot be explained by historical and strategic conditions of the trial alone. One obstacle is the high work load among clinicians, which limits their ability to actively participate. A second obstacle may be ambivalence and hesitance towards working within a therapeutic manual over a longer period of time. We have received feedback from some clinicians who feel deprived of clinical autonomy when working within the defined CAMS framework. Other therapists have acknowledged that although a patient may satisfy the inclusion criteria, they refrain from recruiting the patient to the project because they consider TAU to be the “best practice or choice” for the eligible patient. Hence, one factor seems to be acceptance of the randomization procedure and of the project aim (to empirically evaluate the effectiveness of TAU versus CAMS), and another is perhaps paternalism, as noted by Howard [51].

As the CAMS approach enforces an endured focus on suicidality over some period of time, the approach might challenge myths about suicidality [1]. One relevant concern may be the belief that suicidal thoughts and behavior worsen if you talk about and focus on them. Another myth that may affect recruitment is the belief that people who are talking about their suicidal thoughts are truly not in danger. Recently, Law and colleagues [52] reported no indication of harmful effects from repeatedly focusing on suicidal risk and assessments, even for at-risk patients with borderline personality disorder.

Working with suicidal patients often evokes difficult feelings and considerations in both therapists and patients. The CAMS approach brings a clear and endured focus towards the core issues of the patients’ suicidal thoughts and feelings up until the problem is resolved. Consequently, a joint awareness is gained towards the subjective suicidal experiences. As these issues are anxiety provoking, both patient and therapist might seek to avoid or minimize the problem. Unconsciously, this focus may be difficult both for the system as a whole and for the clinicians (e.g., there may be concerns about extended stay at the inpatient clinic if one focuses on the patient’s problems). These factors combined may be at play as we experience recruitment difficulties.

Strategies to meet these challenges are again “a hands on” presence in the clinics and at strategic meetings, in addition to persistence and ongoing dialogue with both clinicians and leaders about current knowledge in suicidology. Sustained communication and commitment throughout the leadership hierarchy from top to bottom in the clinical system are important to ensure genuine cooperation and partnership.

Despite these considerable challenges, we do consider the trial to be feasible and realistic.

### Selection bias after all?

One may question whether our patient population is preselected. At the outset, all patients who present with moderate to severe suicidal thoughts, plans, and behaviors are eligible to participate in the project. However, most suicidal patients receiving treatment in our clinic are not invited to participate. An array of factors may explain why, and in the end may serve as a selection bias. Recruitment difficulties as discussed above may be one factor. A low general capacity or opportunity to include new patients and perform the randomization procedure is another. The study’s low representation of therapists at each of the participating departments and sites in our services limits the rate of inclusion. One could ask whether a particular group of well-functioning patients is more often invited to participate and more eligible to accept attendance in the research project. Moreover, as this study demands an informed consent to participate, those with the more severe forms of depression and psychosis are not included.

Even though our inclusion criteria are wide, our services as a whole do not offer treatment to all patients who are referred. One group who might be denied treatment within our facilities consists of people who suffer from suicidal problems and ideation but who are considered not to have the “right to necessary health care,” a judicial term and principle that guides selection of those who are to receive treatment within specialized care in Norway. This declined group may include people with chronic psychiatric and psychosocial problems with multiple previous unsuccessful treatment attempts. This group is usually referred to local community health centers.

Another group who does not receive treatment is those who for different reasons decline treatment and follow-up from the specialized health care system after a suicide attempt.

One subgroup seems to trigger objections among the clinicians when it comes to recruitment to the study: the patients assessed as “not truly suicidal” or those “crying for help.” Patients with recurring or chronic suicidal thoughts combined with non-severe suicide attempts and multiple (failed) treatment trials are commonly regarded as “chronic” and difficult to treat effectively. However, DBT has been shown to be effective in reducing symptom severity and suicidal ideation in this group [11]. Since suicide is an infrequent incident, and known risk factors are insensitive and unspecific, true prediction of suicide is hardly possible and always includes false positives, as shown in a classic study by Pokorny [53]. Assessing suicidal risk and treatment of patients with suicidal risk is a multifactorial process that in most instances is very difficult to conduct. Clinical judgments and assessments are influenced by factors such as general experience, training, competence skills, belief systems, cultures, and perhaps myths about suicidality as noted above. However, current evidence documents that people with psychiatric disorders, previous inpatient stays, previous suicide attempts, and suicidal ruminations are at risk [54]. Thus, it seems important not to exclude patients assessed as false positives based on clinical judgment, as such judgments are fallible. We meet this challenge by leading an ongoing dialogue with the therapists out in the clinical units, repeating and reminding them about the inclusion criteria. Presence and attendance in multiple fora are important, from formal meetings to more sociable lunches.

## Trial status

The trial opened by recruiting the first patient in February 2015 and is still recruiting. We aim to include 100 patients to the project and will end inclusion by autumn 2016.

## Abbreviations

AUDIT: Alcohol Use Disorders Identification Test
BSSI-C: Becks Scale for Suicide Ideation - Current
CAMS: Collaborative Assessment and Management of Suicidality
CIDI: Composite International Diagnostic Interview
CRS: CAMS (adherence) Rating Scale
CT-SP: Cognitive therapy for suicide prevention
DBT: Dialectical behavior therapy
DUDIT: Drug Use Disorders Identification Test
GSE: Generalized Self-Efficacy Scale
ITT: Intention to treat
MINI: Mini International Neuropsychiatric Interview
NPR: National Patient Registry
OQ-45: Outcome Questionnaire - 45
RCT: Randomized controlled trial
SASIC: Suicide Attempt Self-Injury Count
SSF: Suicide Status Form
TAU: Treatment as usual
WAI-SR: Working Alliance Inventory - Short Revised
WHO: World Health Organization
